# The Effect of Feedback on Surgeon Performance: A Narrative Review

**DOI:** 10.1155/2020/3746908

**Published:** 2020-10-19

**Authors:** Kelsey A. Rankin, Jordan Brand, Daniel H. Wiznia

**Affiliations:** Yale School of Medicine, 47 College Street, New Haven, CT 06510, USA

## Abstract

Surgeons play a critical role in the healthcare community and provide a service that can tremendously impact patients' livelihood. However, there are relatively few means for monitoring surgeons' performance quality and seeking improvement. Surgeon-level data provide an important metric for quality improvement and future training. A narrative review was conducted to analyze the utility of providing surgeons direct feedback on their individual performance. The articles selected identified means of collecting surgeon-specific data, suggested ways to report this information, identified pertinent gaps in the field, and concluded the results of giving feedback to surgeons. There is a relative sparsity of data pertaining to the effect of providing surgeons with information regarding their individual performance. However, the literature available does suggest that providing surgeons with individualized feedback can help make meaningful improvements in the quality of practice and can be done in a way that is safe for the surgeons' reputation.

## 1. Introduction

In order to continuously improve surgical quality and outcomes, it is important to have concrete metrics to compare individual surgeon performance. Given that surgical skills are not equivalent across all providers, differences in ability contribute to variation in outcomes following surgical procedures. It may come as no surprise that recent studies have demonstrated a correlation between surgeon skill and patient outcomes, which may be a motivator for surgeons to continuously strive to improve surgical prowess [1–3]. Although many different strategies for tracking and reporting surgical outcomes exist, there is a paucity of literature exploring the utility of measuring individual surgeon performance data and the impact it has on quality improvement. In this review, we discuss different strategies for organizing and reporting individual physician data, as well as the impact such tools have on surgeon improvement and clinical outcomes.

## 2. New Contribution

Despite the increasing emphasis on quality improvement in healthcare delivery, there remains to be a significant body of research or interventions assessing individual surgeon performance and outcomes. This narrative review seeks to provide a holistic framework to approach the idea of individual-level feedback. The articles selected and used to contribute to this review were based off literature from pediatric, cardiothoracic, general, orthopedic, robotic, surgery, urology, cardiothoracic surgery, general surgery, orthopedic surgery, robotic surgery, and other quality improvement journals. Polls of existing surgeons cite a plethora of reasons to discourage this analysis, but the few studies where this has been conducted have shown improvements in outcomes based on the availability of this data. These conflicting views posit the necessity for a comprehensive review of all surveys of attitudes, interventions, and suggestions on this contentious topic into one place. A narrative review structure will allow us to synthesize available data, critique existing platforms, and provide possible suggestions for future implementations. Quality improvement is of utmost importance to policymakers and key stakeholders, and this review represents a centralized, condensed collection of surgeon polls on the institution of individual feedback, means of collecting surgeon-specific data, suggested ways to report this information, pertinent gaps in the field, and the results of giving feedback to surgeons. The contents of this review provide suggestions for means of collecting this information in a productive manner to both surgeons and patients, and the positive implications of their integration into clinical practice at large scale.

## 3. Methods

### 3.1. Data Sources

A PubMed literature review was performed using key phrases such as “surgeon feedback” (2,748), “surgeon dashboard” [4], “surgeon performance” (91,768), “surgeon scorecard” [5], and “surgeon evaluation” (69,858). This search was performed iteratively to monitor any newly published resources, through April 2020, and captured all research conducted prior to this date.

### 3.2. Inclusion Criteria

1. Study must pertain to surgeon quality improvement
2. Study must concern individual-level feedback
3. Study must look at the effect of collecting and reporting data, rather than method of collection
4. Studies were in English

### 3.3. Data Extraction

Relevant studies were determined using the aforementioned inclusion criteria. Many quality improvement studies appeared in our search that did not directly pertain to this topic. First, abstract information was discerned, followed by full article review to determine applicability. Articles were reviewed by two authors (KR and JB) to determine relevance. Throughout the review process, article references were reviewed in order to expand the relevant data collection. Additionally, further PubMed searches were performed as themes within the currently presented review were developed. After final review, a total of 46 articles were identified as pertinent to this review.

## 4. Results

### 4.1. Perceived Barriers to Instituting Surgeon-Level Feedback

Today, suspicion and cynicism are some of the biggest obstacles to instituting a dashboard [6]. Surgeons have significant apprehensions regarding collecting outcome data for the purpose of either public reporting or institutional quality improvement projects [6, 7]. As described by Jenkins and Cooper, these apprehensions include the potential consequences of publishing disparaging data, promoting risk-averse behavior, misrepresenting data, creating a ceiling effect, and eroding intrinsic motivation [8]. In one questionnaire of cardiothoracic surgeons, 90% of participants believed that individual data should be collected, but not made public, and 69% believed mortality data should only be reported on a hospital-wide basis and not on the individual surgeon level [7]. Complicating this fraught topic is US healthcare's capitalist nature, where surgeons must compete in a marketplace. These quality metrics could have impacts on their marketability and patient volumes.

One such concern is that data shared publicly may affect patient referral patterns. Brown et al. explored the impact of publicly reported outcome data on referral rates to cardiac surgeons [9]. Cardiothoracic surgery has a long history of sharing quality outcomes with the public [10]. However, after two decades of data collection and public reporting, surveyed cardiologists stated they do not discuss with patients the scores of surgeons to whom they are referring. Additionally, studies suggest that most patients do not incorporate the reported outcomes into their decision in choosing a surgeon [11, 12]. For example, the Pennsylvania Health Care Cost Containment Council regularly publishes and distributes risk-adjusted mortality rates of Pennsylvania cardiac surgeons in its Consumer Guide to Coronary Artery Bypass Graft Surgery. Despite this publicly available information, only 1% of patients undergoing cardiac bypass were aware of their surgeon's mortality statistics [13]. To our knowledge, there are no prospective studies exploring how referrals and new patient visits change in relation to publicly available outcome reporting. Additionally, it is difficult to determine the frequency at which patients independently sought cardiac surgeon's performance scores prior to seeking consultation. Additional qualitative or quantitative data on referral practices and patients' decision making based on outcomes reporting would be beneficial, but to date financial concerns surrounding the influence of reporting on patient referrals have not been demonstrated, and there lacks an evidence-based argument against surgeon-level reporting.

Fostering surgeons to exhibit risk-averse behavior is another commonly cited reason to refrain from tracking individual surgeon data [8, 14–16]. According to Jenkins and Cooper, this topic is the most frequently cited issue pertaining to negative consequences of publishing surgeon-specific data. They define risk aversion behavior as when a “surgeon chooses not to operate on a patient perceived to be at risk of a poor outcome in order to improve results.” They also suggest that risk aversion reduces innovation [8]. In a study published by Jarral et al. in the United Kingdom (UK), 86.6% of surveyed cardiothoracic surgeons believed that monitoring surgeon-specific mortality data has led to risk-averse behavior [14, 17]. They also noted less-experienced surgeons are likely to share this opinion, and those playing an active role in tracking the mortality data were less likely to share this view. The reason less-experienced surgeons have a negative view on surgeon-specific outcome reporting is unclear. One potential reason may be that less-experienced surgeons tend to take more call shifts in order to build their practice and may therefore encounter a greater number of emergent cases [18]. In these scenarios, mortality data does not reflect the difference between an inability to rescue from death versus death due to surgeon error. Additionally, publicly reported outcome data creates an additional layer of risk taking, and more experienced surgeons may be more accustomed to taking risks [14]. Regardless, a majority of correspondents (74.7%) felt that trainee experience was harmed due to monitoring surgeon-specific mortality data, perhaps because surgeons are less likely to give independence to trainees [14].

Though there is propensity to cite risk aversion as an argument against collecting surgeon-specific outcome data, it is difficult to determine whether this concern translates to real-world human behavior. Dr. Maggard-Gibbons notes that early reports after implementing the New York Cardiac Surgery Reporting System (CSRS) suggested that high-risk cardiac patients were being diverted to the Cleveland Clinic [15]. However, she notes that a few years later, more comprehensive evaluations failed to reveal such practice patterns. Similarly, a UK study found that the number of abdominal aortic aneurysm (AAA) surgeries performed increased after the implementation of surgeon outcome reporting without significant change in patient factors such as age, aneurysm size, and results of cardiopulmonary exercise testing, suggesting that reporting outcomes did not affect surgeons' willingness to take on more difficult cases [16].

However, it has been shown that there are discrepancies in partitioning of high-risk patients. Looking at six surgeons within one institution, Ferraris et al. found that 83% of deaths occurred within the highest quartile for risk. One surgeon had significantly more high-risk patients (*p* < 0.05), demonstrating a shunting of high-risk patients and an inordinate risk burden on select physicians for unmeasured reasons [19].

These aversions and concerns surrounding both the collection of and dissemination of surgeon-level feedback are of paramount importance and also shed light on the multifactorial potential for impact of this feedback: as a didactic tool for trainees and experienced surgeons to improve outcomes, as well as a publicly reported tool that influences surgeon/institution share of the market. It is important that future studies evaluating the impact of surgeon-level feedback attempt to tear apart these consequences.

### 4.2. Current Gaps

Several studies have tried to use existing high-quality databases to evaluate individual surgeon performance. Using National Surgical Quality Improvement Program (NSQIP) data, many studies found that, in order to determine surgeon-level performance and reliability metrics for specific outcome parameters, the volume of cases needed for analysis with >80% power was significantly higher than the available number of cases [20, 21]. Using 30 months of NSQIP data from 51 hospitals in Illinois, analyzing 2,724 physicians, Quinn et al. found low surgeon-level variance across all 7 outcome measures (0.007–0.074) and low median reliability (0.1). The authors' takeaway is even given a high granularity of detail, and the current reporting of surgeon-level outcome metrics deeply limits the ability to discern any real individual-level differences [21].

Many studies argue for the necessity of surgeon-level analysis and reporting [22, 23]. The ProPublica scorecard was one such tool designed in hopes of tracking surgeon performance, but significant design flaws have wrought concern regarding this particular modality [24–27]. The ProPublica surgeon scorecard is a privately owned, publicly accessible database looking at mortality and complication rates at the surgeon-level for eight elective procedures within the Medicare population [5]. Some acknowledge its flaws while positing for its continued use with many revisions [24, 26]. The overwhelming critiques focus on the chosen factors contributing to their reported “adjusted complication rates” (any hospital readmission or death within 30 days of surgery) and the factors that are missing (complications without readmission, any complications >30 days, complications during the index hospitalization). The authors argue for the amelioration of this tool, as it represents an opportunity to drive quality improvement and aid in patient selection of provider [22, 24, 28].

### 4.3. Developing Tools for Surgeon-Level Feedback

Outcome tracking, evaluation, and improvement is a frequent topic of conversation throughout modern medicine [4, 29]. Many arenas of medicine have created tools to track specific metrics and measure improvement; modeling surgeon-specific outcome tools after these feedback tools may enhance development and efficiency. For example, Fox et al. reported a pediatric medicine dashboard used to measure comparable metrics across four different departments. After the researchers shared the data, they saw increased timeliness of discharges, hospital committee participation, and grant funding [30]. The authors also offered several suggestions for developing outcome feedback tools:

1. Choose common metrics that can easily be tracked
2. Include multiple domains and multiple metrics to create a clear picture of one's activities
3. Confirm common definitions of metrics amongst subjects
4. Set realistic expectations regarding the number of subjects and administrative effort involved in data collection
5. Integrate data collection into the electronic medical record
6. Take a holistic approach to analyzing complete data rather than focusing on individual metrics
7. Have a plan for sharing data with hospitals and administrators [30]

In a novel model focused on improving objectivity of surgeon-specific feedback, Hung et al. look at the use of a da Vinci Systems device to collect automated performance metrics during robotic surgery. By applying machine learning, the authors propose a more objective collection of performance data aimed to assess surgeon proficiency and illuminate discrepancies. The study demonstrated that more expert surgeons use their dominant hand more than their more novice counterparts, in contrast to previous claims that bimanual dexterity is the “ideal surgical trait” [31]. The technique was also able to identify specific camera manipulation parameters that correlated with surgical expertise and better outcomes. The overall takeaway is that these more objective measurements of surgical performance in robotic surgery could be mobilized to enhance patient outcomes and provide an additional safety check for patients prior to operation [32].

### 4.4. Data Collection and Distribution

Shahian et al. state that cardiothoracic surgery is the most well-studied surgical specialty with regard to quality improvement. They made several recommendations for developing guidelines, such as focusing outcome measures on common procedures, using quality scores that consider structure, process, and outcomes, exploring preoperative, intraoperative, and postoperative domains, and measuring factors that should be interpretable and actionable by providers [13].

In 2015, Shahian et al. reported the composite score system his institution had used to provide feedback to surgeons. This composite score compared surgeons to a single standard score, as opposed to directly comparing surgeons to one another within a department [33]. A composite score may help facilitate surgeon growth without fostering unnecessary competition amongst colleagues. An additional strength of this method is that it used a weighted risk assessment, enabling a surgeon's most frequently performed procedure to have the greatest effect on their composite score. One downfall to the composite score used in this report, however, is that it did not break down the relative subcomponent scores, which contributed to the composite score, thus diminishing the opportunity to identify specific areas for improvement [33, 34].

As discussed by Fiala, perception of quality of service is composed of both technical quality and functional quality components [35]. In Fiala's article, functional quality is defined as the manner in which services are delivered to customers and represents how the customer experienced the human interactions that occurred during the care process. When rating their healthcare experiences, patients appear to have greater interest in the functional quality of care rather than the technical component [35]. Given that much of the available literature regarding individual surgeon assessment is related to objective outcomes and technical factors; perhaps it should come as no surprise that patients' have shown little interest in individual surgeon scores. This presents an opportunity to contribute potentially patient-actionable data to the field.

In a review by Radford et al., several concerns were raised regarding the effects of publishing surgeon-level outcome data to the public. As this type of data continues to become publicly available, questions regarding data quality, patient use and interpretation, trainee experience, and risk adjustment will continue to rise [36]. Prospective studies will be required in each of these categories to elucidate the results of data publication.

### 4.5. Adjustments for Interpretation of Data

In a 2015 survey, Jarral et al. indicated that cardiothoracic consultants had concerns that published outcome data stood the risk of being misinterpreted by potential patients [14]. Surgeons concerned with data misinterpretation may be put at ease if patient risk stratification and comorbidities are taken into account when analyzing outcome data. Beck et al. discussed the importance of this precaution in their study of more than 8,000 patients from 51 hospitals. They found a wide variety of mortality and complication rates amongst different facilities but noted that there was also a wide variety in case complexity amongst hospitals. Thus, they suggest that, in order to develop care benchmarks or measure hospitals' ability to meet set standards, one must perform case mix adjustments [37]. Physicians who take on more complex cases are expected to have more complications, which should be taken into consideration when evaluating and comparing individual outcomes.

Nonetheless, it may prove difficult to present data to patients in a clear and concise manner. In a questionnaire by Sathianathen et al., certain populations expressed a willingness to incur greater out-of-pocket expenses in order to be treated by a particular surgeon, even though the hypothetical surgeons were statistically equivalent [38]. The authors conclude that data misinterpretation poses a financial risk to patients. Another prospective questionnaire showed that a majority of patients themselves believe they are likely to misinterpret data. This same survey showed that patients prefer access to hospital-level data over surgeon-level data and weigh the patient-physician relationship more heavily than individual outcome performances [39]. These findings do not necessarily indicate that surgical outcome parameters should not be tracked, but they highlight the need to exercise significant caution with how and if the data should be made available to the general public.

### 4.6. Effects of Providing Surgeon-Specific Feedback

Individual-level performance evaluation can provide hospital leaders and patients with a powerful tool to improve patient safety and outcomes following surgery [23]. Surgeon-specific feedback has demonstrated improved surgical technique and outcomes. For example, one of the few studies to date exploring how outcome tracking at the surgeon-level improved surgical technique comes from the urology literature. In this study, surgeons received comparative feedback on their rate of positive surgical margins after radical prostatectomies. The authors looked at the effects of providing comparative performance measures and its ability to decrease the rate of positive surgical margins. They found that urologists' surgical margins improved after they had received “report cards” every six months over a one-year period. The data was compared to their own self-matched data, deidentified data of their colleagues, and institutional aggregate data from the study period. Interestingly, the five surgeons with positive surgical margin rates higher than the aggregate department rate in the preintervention period showed improvement after intervention [40]. This study may foreshadow future studies aimed at improving surgeon performance.

A similar study by Mabit et al. sought to explore the utility of surgeon-specific feedback in decreasing surgical site infection (SSI) occurrences. They developed a surveillance system that tracked SSIs across a hospital system and provided report cards to surgeons. This study demonstrated that this surveillance system reduced the incidence of SSIs, particularly in orthopedic traumatology [41]. This is not a universal finding, as other studies have demonstrated that surveillance of SSIs with surgeon-specific feedback failed to reduce the incidence of SSIs over their study period [42]. The discrepancy between these studies reinforces the necessity for continued research into the utility of surgeon-specific feedback.

Dashboards also serve to increase surgeon awareness of their costs and have demonstrated cost improvements since rollout. For example, Tabib et al. also looked to improve efficacy of healthcare spending by reducing operating room costs with real-time, surgeon-specific feedback on procedure expenses [43]. In this study, urologists successfully reduced operating room cost using immediate feedback and cost comparisons. Individuals' performances were identifiable amongst colleagues, and this transparency enabled collaboration in identifying areas for cost reduction and implementing appropriate changes. Tabib concluded that the transparency in their methods successfully altered surgeon behavior but noted that further studies are necessary to show equivalence in patient outcomes [43].

In another example of cost improvement, Robinson et al. looked to improve the value of care in pediatric appendectomies through a surgeon-specific approach [44]. In this prospective study, an automated dashboard for surgeon's operative expenditures was created, and monthly reports were generated for each physician. After six months of reporting, this institution experienced a decrease in supply costs. This came without any significant change in procedure duration or adverse events (defined as 30-day return to the operating room, interventional radiology drainage, surgical site infection, or readmission) [44].

Winegar et al. examined the effects of using a monthly, unblinded surgeon scorecard on economic effects and patient outcomes for patients undergoing total joint arthroplasty (TJA) at a tertiary hospital [45]. Metrics from the first scorecard issued were compared with those on the tenth scorecard given to surgeons. The mean cost of TJA decreased 8.7%, largely attributable to a decrease in mean total direct variable costs, which are under control by the surgeon. Additionally, patients' length of stay decreased by a mean of 0.2 days. There was also an improvement in home-discharge rate as well as a decrease in 30- and 90-day readmission rates, though these variables did not reach statistical significance. Nonetheless, these findings suggest that surgeons are able to decrease the economic burden of TJAs without detrimental effects on the quality of patients care [45]. These findings are consistent with previous literature that demonstrates that scorecards can improve operative cost efficiency with equivalent or improved patient outcomes [46].

## 5. Conclusions

Surgeon-specific outcome data offers an opportunity for surgeons to identify and address potential areas for improvement. The potential for risk-averse behavior, data gaming, financial consequences, and interference with trainee education are valid concerns, but the limited literature available has not demonstrated fruition of these fears. Surgeon-level data is an important metric to drive quality improvement and inform patients surrounding their potential physicians [26]. As more data becomes available on the potential value of monitoring surgeon-specific outcomes, the debate concerning publicizing surgeons' data will become increasingly relevant. The limitations of this study arise from the paucity of literature looking into individual-level feedback and the possibility that some studies used terms outside the aforementioned key terms. Future studies should examine the utility of specific feedback tools such as dashboards and scorecards, the effect of data collection on surgeon behavior, and the consequences of feedback on patient outcomes.

## Data Availability

No data were used to support this study.
